# Understanding the Relationships Between the Consumer Perception on Food Risks, Quality, and Safety Indicators of Braised Meat Sold in “Dibiterie” Restaurants in Dakar, Senegal

**DOI:** 10.3389/fvets.2021.788089

**Published:** 2021-11-26

**Authors:** Malik Orou Seko, Walter Ossebi, Nibangue Laré, Bassirou Bonfoh

**Affiliations:** ^1^Ecole Inter-Etats des Sciences et Médecine Vétérinaires, Dakar, Senegal; ^2^Centre Suisse de Recherches Scientifiques en Côte d'Ivoire, Abidjan, Côte d'Ivoire

**Keywords:** dibiterie, meat, perception, quality, safety, risk, structural equation model, Senegal

## Abstract

Dibiteries are restaurants that sell braised meat of small ruminants and sometimes chicken. Current microbiological data indicate that the products sold are sometimes contaminated with pathogenic microorganisms exceeding the quality standards recommended for human consumption, hence a real public health concern. Despite the lack of hygiene, these establishments continue to thrive in the Senegalese food ecosystem. However, very few studies have analyzed the socio-economic motivations and risk representations of these populations who participate in the growing demand for meat from dibiteries. The main objective is to understand the relationships between consumer perception of food risks, quality, and safety indicators of braised meat sold in Dibiteries in Dakar. A total of 479 people from 404 households in the Dakar region were randomly selected and surveyed on the consumption of dibiterie meat using a structured questionnaire. The questionnaire allowed to measure the relative importance given by each interviewee to the indicators related to the risk of food infection, and the quality and safety of dibiterie meat. The structural equation model was used to design the paths and analyze the relationships. Of the 479 people interviewed, 291 people consumed dibiterie meat. Only 16% of consumers strongly perceive the quality and safety of meat. This strong perception has been positively associated with monthly food expenditure, while the age of consumers explained it negatively. Among the latent variables identified, the perceived price effect and the dibiteries' expertise were positively related to the perception on the safety and the perception on the nutritional quality of the product. The nutritional quality of the product had negatively impacted the risks of food infection perceived by consumers. The results of this study suggest the strengthening of hygiene standards in dibiteries and the awareness of consumers, especially young people, about the potential health risks associated with the consumption of dibiterie meat. Further work on willingness to pay to improve the safety of dibiterie meat is needed.

## Introduction

Animal source foods are essential for the nutrition and livelihoods of low-income populations in sub-Saharan Africa. In low- and middle-income countries (LMICs), they contribute significantly to diets. The demand for fish, milk, and meat will continue to grow over the coming decades, thanks to population growth and changing consumption practices linked to urbanization and rising incomes.

Meat is an important element of the daily diet for much of society and is considered as a valuable food from a nutritional point of view (1). Indeed, meat provides important nutritional elements including proteins, fats, vitamins, and minerals that effectively contribute to the normal functioning of consumers' bodily systems (2). Although the benefits of meat consumption are significant, meat is a highly perishable product and can often cause food poisoning in consumers due to poor conditions of transport, storage, processing, or marketing. Therefore, the monitoring of food safety risks across all animal production chains (from stable to table) is of great interest. In addition, a diet rich in meat can also have a potential negative effect on human health due to the high content of cholesterol and saturated fatty acids that may be contained in meat (3). For red meats, such as beef, mutton, and pork, studies have associated a reduction of their consumption as a reflex response linked to the individual perceptions of health risks (4, 5). The levels of cholesterol and saturated fat in red meats have been reported as specific health factors influencing consumer choices (6, 7).

Meat consumption habits are unpredictable due to the constant changes in consumer behavior toward meat and other food products (8). For consumers to voluntarily buy and consume a particular meat product, their perception of it must be positive. If consumers have a negative perception of a meat product, their purchasing behavior will be negatively affected (9). Consumer behavior toward food, especially meat, is characterized by changing preferences (10). Indeed, food choice is a phenomenon resulting from the interaction between a variety of factors (11). Thus, consumers consider several characteristics to determine food product acceptance, sensory characteristics, nutritional value, convenience, and its impact on their health (12, 13). Indeed, in addition to the price of the product frequently targeted by consumers, factors such as appearance, convenience and perceived quality as well as safety (14, 15), social, individual, economic, and cultural aspects influence decisions taken on the market place (8). Thus, consumers now require safe and good quality food products at a reasonable price (15). Therefore, understanding consumer behavior becomes vitally important, as the way in which consumers' expectations are met decisively influences their purchasing decisions (16, 17).

In sub-Saharan Africa, the food processing and marketing link dominated by catering is growing rapidly, particularly in the informal sector where animal source products are sold at affordable prices and highly appreciated especially by populations with low income. However, the technologies and processes applied in these markets by often unskilled food handling personnel make the finished products unfit for human consumption. This is the case in Senegal with small food processing units operating in the informal sector, called “Dibiterie.” These restaurants offer braised meat of small ruminants and sometimes chicken for human consumption. According to current evidences, the products from these restaurants are sometimes contaminated with pathogenic microorganisms exceeding the quality standards recommended for human consumption (18, 19), hence a real public health concern. This situation is linked to the non-application of certain measures of good hygiene practices by the staff. Despite the lack of hygiene, these establishments continue to thrive in the food ecosystem, thus attesting to the growing demand for these products by the Senegalese populations, whose motivations are multiple. However, very few studies have analyzed the socio-economic motivations of these populations who participate in the growing demand for dibiterie meat. In addition, the representations of the risks associated with the consumption of these products have not yet been clarified. The main objective of this study was to understand the relationships between consumer perception of food risks, quality, and safety indicators of braised meat sold in Dibiteries in Dakar. Specifically, this involves (i) characterizing the levels of perception on the quality and safety of dibiterie meat; (ii) identifying the factors associated with levels of perception of the quality and safety of dibiterie meat; (iii) assessing the relationships between the variables associated with the perception of the quality and safety of dibiterie meat and their impact on the perception of the risk of food infection; and (iv) determining the representations of the risks associated with the consumption of dibiterie meat.

## Materials and Methods

### Study Area and Target Population

This is a descriptive cross-sectional study that was carried out from November 2018 to February 2019 among consumers of dibiterie meat in households and dibiterie tenants in the Dakar region in Senegal. This region consists of the departments of Dakar, Guédiawaye, Pikine, and Rufisque. This framework of investigation was chosen because the department of Dakar is the main pole of demand for products of animal source food due to the large share, i.e., 23% (3,529,300 inhabitants) of the population of Senegal, it concentrates (20). In addition, consumers who reside there have a higher purchasing power compared to other regions. However, the suburb of Dakar represented by the departments of Pikine, Guédiawaye, and Rufisque brings together ~63% of the region's population. In addition, the department of Pikine is home to the Dakar region slaughterhouse. The management of this slaughterhouse is ensured by the Société de Gestion des Abattoirs du Sénégal (SOGAS). The department of Pikine is therefore a crossroads for households and tenants of dibiteries in search of good meat quality for human consumption.

### Sampling and Sample Size

Household sampling was performed using the simple random method and the sample size *n* was estimated using Thrusfield's formula (21):


(1) $$n=\frac{\left[Z^{2}*p(1-p)\right]}{d^{2}}$$

with *n* = the number of households to be surveyed; *Z* = 1.96 (confidence level deduced from the 95% confidence rate); *p* = 50% (expected prevalence of households consuming dibiterie meat); *d* = 5% (margin of error).

The sample size is 384 households. In order to have the maximum number of dibiterie meat consumers, the choice of neighborhoods was made in a reasoned manner and based on the distribution of dibiterie establishments in the Dakar region. Indeed, Orou Seko (22) found that the dibiteries are mainly located in popular neighborhoods in the Dakar region. Thus, the first step was to investigate the popular neighborhoods covered by this study. High-income neighborhoods have been associated to these different popular neighborhoods. Knowing that income strongly determines the purchasing power and type of housing of households, the neighborhoods to be sampled were first divided into three groups according to a classification adopted by Mankor (23) associated with the results of the study by Orou Seko (22). These are low-income popular neighborhoods, middle-income popular neighborhoods, and high-income neighborhoods. Based on the income level and the housing type of the neighborhoods, a random draw was made to obtain representative neighborhoods of the three groups and the sample size was proportionally distributed over all the selected neighborhoods.

Within each neighborhood, the choice of households and people to be surveyed was made randomly and according to their availability and willingness to answer our questions. In order to avoid gender bias, three members within each household—a man, a woman, and a young person (man or woman)—were surveyed. Inclusion of people was based on the following criteria: (i) individuals of both sexes who had agreed to participate in the investigation by signing the informed consent form; (ii) persons aged at least 16 years who have obtained the consent of one of the parents or a member of the family. At the end of the investigations, 478 people including 291 consumers of dibiterie meat were surveyed. The distribution of this size by gender shows a non-significant difference, i.e., 215 men (45%) and 263 women (55%). The socio-economic and demographic profile of the sample of dibiterie meat consumers is presented in Table 1.

**Table 1:** Socio-economic and demographic profile of consumers of dibiterie meat in households of the Dakar region (*n* = 291).

**Characteristics**	**Modalities**	**Frequency**	**Percentage**
Gender	Male	141	48
	Female	150	52
Age (years)	16–20	15	5
	20–40	173	59
	40–60	69	24
	≥60	26	9
	Non-respondent	08	3
Ethnic group	Wolof	80	28
	Sérère	33	12
	Peulh	60	20
	Lébou	47	16
	Djola	18	6
	Other Senegalese ethnicities	20	10
	Non-Senegalese ethnicities	33	8
Religion	Muslim	262	90
	Christian	29	10
Marital status	Young	10	3
	Single	105	36
	Married	152	52
	Widower	11	4
	Divorced	13	5
	Non-respondent	00	0
Level of education	Without formal education	18	6
	Primary	78	27
	Secondary	91	31
	University	91	31
	Koranic	11	4
	Non-respondent	02	1
Socio-professional category	Public servant	22	8
	Employee	36	12
	Manual-workers	45	15
	Trader	38	13
	School-boy/Student	57	20
	Housewife	58	20
	Retired/Unemployed	13	4
	Other professions	16	6
	Non-respondent	06	2
Monthly food expenditure (FCFA*)	<25,000	07	3
	25,000–50,000	27	9
	50,000–75,000	27	9
	75,000–100,000	37	13
	>100,000	164	56
	Non-respondent	29	10
Monthly income (FCFA*)	<50,000	19	6
	50,000–100,000	46	16
	100,000–150,000	30	10
	150,000–200,000	31	11
	>200,000	119	41
	Non-respondent	46	16

* *FCFA, Franc de la communauté financière africaine (1 USD = 565.1686 FCFA, https://fr.exchangerates.org.uk/convertir/USD-XOF.html)*.

Moreover, at the Dakar slaughterhouse located in the department of Pikine, six meat consumers (men and women) and two dibiterie tenants were selected, respectively, for a focus group discussion (FGD) and semi-structured interviews.

### Theoretical Framework and Study Design

Rapid economic development and recent changes in the food supply chain have contributed to increased interest in the issues of quality and safety in the food sector. In the minds of consumers, the notion of the quality of a food product appears to be closely linked to the perception of its safety. A study investigating the relationship between food quality and safety has found that people seem more prone to regard a food product as safe if they consider it to be of high quality rather than the opposite (24). Several studies have highlighted the fact that the definition of quality is not unified but rather depends on the different perspective from which it is evaluated: a definition in technical and production terms may differ from the perception of consumers (25). From the point of view of consumers, in fact, several aspects help to define the quality of a food product: these are not only intrinsic qualities such as taste and other organoleptic properties, but also external factors such as origin and labeling (26, 27).

The quality theory based on the information economics approach to user-oriented quality was used for the design of this study (28). Indeed, consumers look for high-quality food products and they infer this quality on the basis of a certain group of indicators, or attributes, which are classified according to the degree of visibility, namely: the search, experience, and credence or belief attributes (29). This approach has been applied to meat by many authors (30, 31). Firstly, there are the search or expected quality indicators and often referred to as “quality cues”—the evaluation of indicators of the nature of the products to be purchased. These attributes can be classified into two types, intrinsic and extrinsic cues. Intrinsic cues, described as visible inherent characteristics of the product, are important in determining quality expectations in many categories of fresh foods. Extrinsic indicators represent information related to the product but which is not physically part of the product, which can be modified externally (31). Secondly, there are experienced quality indicators that can only be revealed after purchasing and consuming the product. However, according to Verbeke et al. (32), consumers expect the experience quality to meet their expectations and, therefore, are increasingly more open to the use of extrinsic cues to support such evaluations. Thirdly, there are indicators of the credence or belief quality—characteristics that persist even after purchasing and consuming the product. Belief quality attributes are those that consumers can never assess with confidence but based on consumers' opinions of the product itself or the producer, even after consumption (29, 31). This involves health and process benefits (which may satisfy moral and ethical needs), and a consumer cannot with any degree of certainty assess or confirm their existence.

Furthermore, the evidence indicates that using certain intrinsic attributes to deduce quality can be dysfunctional (33, 34). According to Henchion et al. (31), this suggests a discord between the expected and experienced quality due to a misconception of certain intrinsic indices. Grunert (35) argues that this is due to displaced reliance on intrinsic quality cues, which may be the result of relatively few extrinsic indices available to support consumer evaluations. Consequently, it undermines consumers' confidence in the sector, increases their uncertainty about quality expectations, and can lead to dissatisfaction (31). In addition, extrinsic cues offer considerable potential to support the consumer quality assessments in light of evolution of purchasing motivations linked to changing demographics, lifestyles, and knowledge, and raising concerns about safety, health and ethical factors (26, 35).

The debate around these themes focused on several aspects of the product: from organoleptic characteristics to health and hygiene safety, healthiness and nutritional qualities at the place of production, and the ethical aspects associated therewith. Based on previous studies conducted on the perception of meat quality and safety in Morocco and Tunisia (36–38), this study identified and assessed 15 variables that can influence consumers' perceptions of quality and safety of dibiterie meat in households. The indicators linked to quality were as follows: taste, smell (after cooking), price, time constraint, proximity, salesperson's expertise, dibiterie name (brand), and dibiterie renown. As for the indicators of the dibiterie meat safety, it was retained: dibiterie hygiene, place of animal slaughter, veterinary stamp, animal slaughter according to the Muslim rite, rich in vitamins, rich in energy, and microbes. For each of these attributes, the consumer had to report his attitude by indicating his degree of attachment to each of the variables on a five-point Likert scale ranging from (1) “strongly disagree” to (5) “strongly agree” on the basis of the answers to the question related to the elements encouraging consumption (for example: I consume the meat of dibiteries for its characteristic smell after cooking?).

Consumers' perceptions on the risks of food infection were also assessed. All four items related to the five keys to safer food from the WHO (39) were used. For each of these items, the consumer had to report his attitude by indicating his degree of attachment to each of the variables on a five-point Likert scale ranging from (1) “strongly disagree” to (5) “strongly agree” on the basis of the answers to the question related to the food infection risks (for example: washing hands before consuming dibiterie meat helps to prevent food infections?).

In the present study, the first step is to assess the relationships between the variables associated with the perception of quality and those related to the perception of the safety of dibiterie meat, and secondly, to determine how these relationships impact the perception of the risks of food infection using the structural equation modeling (SEM) approach. This approach was used because it allows to (i) specify and test the whole theoretical or conceptual model to determine in what extent the hypothetical model is consistent with the data; (ii) specify and test in the theoretical model more complex paths (i.e., direct and indirect) between variables; and (iii) incorporate latent variables with multiple indicators, while regression analysis would not have allowed the inclusion of several indicators (40).

### Data Collection

The collection of information from households was carried out by administering a structured questionnaire in French or Wolof (local language) at home. The data collected concerned (i) the socioeconomic and demographic characteristics of the interviewees; (ii) indicators linked to the quality and safety of dibiterie meat; and (iii) the perception on the risks of food infection linked to the consumption of dibiterie meat.

The different information was collected through direct or indirect interviews depending on the level of formal education of the participant. Indeed, we sometimes used the service of an interpreter for the translation from the French language into Wolof when the people interviewed did not understand French.

In order to analyze the perceptions and social constructions of risk, an FGD and semi-structured interviews were also carried out, respectively, with the buyer-consumers of meat and the dibiterie tenants within the Dakar slaughterhouse in Pikine department.

### Statistical Data Processing and Analysis

The investigation data were entered using Sphinx Plus2 version 5 software and transferred to the Microsoft 2016 Windows Excel spreadsheet. SPSS Statistics and SPSS AMOS version 23 software were used for statistical analyses of the data. Means followed by standard deviations were estimated for quantitative variables, while percentages were measured for qualitative data.

Meat quality index (MQI) estimation allowed to characterize the levels of consumer perception on the quality and safety of dibiterie meat. The MQI is an “additive index” allowing to measure the relative importance given by each interviewee to the quality of meat through their attachment to each attribute. From this index, different levels of perception of the quality and safety of dibiterie meat were identified. The groups of perceptions selected are subjective and based on the relevance of the expected results. The values of the index range from a minimum of 0 to a maximum of 1 (41). The following equation shows the formulation of the MQI.


(2) $$MQI_{i}=\;\frac{\sum_{s=1}^{m}a_{is}\;*\;X_{s}}{aX}$$

where *a*_*is*_ an integer score given to an attribute (*X*_*s*_) by interviewee *i* (*i* = 1, 2, …, *n*) according to the Likert scale chosen; *s* is the number of attributes (*s* = 1, 2, …, *m*); and *aX* is the maximum potential score that can be obtained by an interviewee (number of attributes multiplied by the maximum score defined by the Likert scale).

Thus, consumers with an MQI >70% are qualified as “strong perception,” while those whose MQI are lower and higher than 50% are qualified as “weak perception” and “average perception.” Multinomial logistic regression (MLR) was then performed to identify socioeconomic and demographic variables that explain the levels of perception on the quality and safety of dibiterie meat. Then, the principal component analysis (PCA) with orthogonal rotation (Varimax) allowed to identify the latent variables characterizing consumers' perceptions on the quality and safety of dibiterie meat using SPSS Statistics software version 23. A latent variable (dimension) was selected and identified if its initial eigenvalue was ≥1. A variable (item) was retained in a component if its absolute initial eigenvalue was >0.3. Using the SPSS AMOS version 23 software, these latent variables were used in a structural equation model (SEM) to identify the different relationships between the variables associated with the perception on the quality and safety and their impacts on the perception of the risks of food infection. A chi-square *p*-value > 0.05 was considered indicative of an exact fit of the model. We have also reported goodness-of-fit indices as measures of approximate fit (42). The following fit indices were used: the root mean square error of approximation (RMSEA), Goodness of Fit Index (GFI), Comparative Fit Index (CFI), and Root Mean Square Residual (RMR). Values <0.05 indicate a good fit for RMSEA. Values close to 0 for the RMR while values ≥0.90 indicate an acceptable fit for the model and data for both the GFI and the CFI (40). Furthermore, on the basis of the model fit indicators, we modified the hypothetical model by removing the paths of the observed variables (items) having standardized coefficients <0.5 (40) and the estimations were recalculated up to obtaining a model that well overall fits to the data. Therefore, several iterations were carried out to arrive at the final model.

Finally, the qualitative information from the FGD and semi-structured interviews were triangulated in order to analyze consumers' constructs on the risks associated with the consumption of dibiterie meat.

## Results

### Levels of Perception on the Quality and Safety of Dibiterie Meat

In order of importance, the decision to consume the dibiterie meat in households was mainly based on the quality and safety attributes such as taste, dibiterie hygiene, salesperson's expertise, dibiterie renown, dibiterie name, and rich in vitamins (Table 2).

**Table 2:** Distribution of the mean scores of the indicators of perception on the quality and safety of dibiterie meat (*n* = 291).

**Category of indicators**	**Items**	**Mean of scores (SD)**	**Cronbach' α**
Dibiterie meat quality	Taste	4.43 (0.89)	0.819
	Smell (after cooking)	4.35 (0.98)	
	Salesperson's expertise	3.58 (1.15)	
	Dibiterie renown	3.13 (1.19)	
	Dibiterie name	3.11 (1.20)	
	Price	2.46 (1.14)	
	Proximity of the dibiterie	2.39 (1.03)	
	Time constraint	2.37 (1.04)	
Dibiterie meat safety	Dibiterie hygiene	3.98 (1.04)	0.679
	Rich in vitamins	3.03 (1.17)	
	Rich in energy	2.86 (1.17)	
	Veterinary stamp	2.73 (1.19)	
	Animal slaughter according to the Muslim rite	2.59 (1.13)	
	Place of animal slaughter	2.47 (1.10)	
	Microbes	2.19 (0.76)	

*SD, Standard deviation*.

The value of the index of quality and safety of dibiteries meat ranged from 0.32 to 0.88. The distribution of this index indicates the existence of three levels of consumer perception according to the relative importance given to the indicators of the quality and safety of dibiterie meat (Table 3). The majority of consumers had a “medium perception” (index between 0.51 and 0.70) of the quality and safety of dibiterie meat (70%). Consumer groups with a “low perception” (index between 0.32 and 0.50) and a “high perception” (index between 0.71 and 0.88) of the quality and safety of dibiterie meat were less represented, i.e., ~15% each.

**Table 3:** Characterization of the levels of perception on the quality and safety of dibiterie meat (*n* = 291).

**Group of consumers**	**Distribution of the quality and safety index of dibiterie meat Limits of variables**		**Number of consumers**	**%**
**Level of perception**	**Minimum**	**Maximum**		
Low	0.32	0.5	43	14.78
Medium	0.51	0.70	203	69.76
High	0.71	0.88	45	15.46
Mean ± SD	0.61 ± 0.09			

*SD, Standard deviation*.

### Factors Associated With the Levels of Perception on the Quality and Safety of Dibiterie Meat

Taking as a reference the group of consumers with an “average perception” on the quality and safety of dibiterie meat, the results of the multinomial logistic regression are presented in Table 4. It emerges that the “low perception” of consumers on the quality and safety of dibiterie meat was positively influenced by the individual monthly income (*p* < 0.01) and negatively by the monthly food expenditure (*p* < 0.05). This means that, compared to the reference group (average perception), people whose monthly income is between 100,000 and 150,000 FCFA have a weak perception of the quality and safety of dibiterie meat. Also, the more people have monthly food expenses of between 50,000 and 75,000 FCFA, the less they tend to perceive weakly the quality and safety of dibiterie meat (compared to the reference group).

**Table 4:** Multinomial logistic regression of factors associated to the levels of perception on the quality and safety of dibiterie meat (*n* = 229).

**Category**	***N* (%)**	**Levels of perception** ^**†**^							
		**Low**				**High**			
		**B**	**SE**	***p***	**OR (95% CI)**	**B**	**SE**	***p***	**OR (95% CI)**
**Location**									
Dakar	146 (64)	−0.06	0.49	0.905	0.94 (0.35–2.50)	0.15	0.49	0.76	1.16 (0.44–3.19)
Suburb	83 (36)			Reference				Reference	
**Age (year)**									
16–25	60 (26)	−1.34	1.31	0.305	0.26 (0.02–3.39)	−2.58	1.18	0.029**	0.07 (0.01–0.77)
26–35	75 (33)	−1.09	1.22	0.374	0.34 (0.03–3.71)	−2.06	1.09	0.059	0.13 (0.01–1.08)
36–45	47 (20)	−0.49	1.27	0.696	0.61 (0.05–7.31)	−1.91	1.13	0.091	0.15 (0.02–1.35)
46–55	20 (9)	−0.88	1.36	0.518	0.41 (0.03–6.00)	−1.83	1.20	0.128	0.16 (0.01–1.69)
56–65	15 (7)	−0.53	1.33	0.692	0.59 (0.04–8.03)	−1.82	1.26	0.150	0.16 (0.01–1.93)
≥66	12 (5)			Reference				Reference	
**Gender**									
Homme	120 (52)	0.36	0.48	0.447	1.44 (0.56–3.68)	0.82	0.46	0.079	2.26 (0.91–5.62)
Femme	109 (48)			Reference				Reference	
**Marital status**									
Not married	110 (48)	0.49	0.51	0.338	1.63 (0.6–4.43)	0.19	0.49	0.69	1.22 (0.46–3.19)
Married	119 (52)			Reference				Reference	
**Formal education**									
Without	19 (9)	−2.13	1.09	0.052	0.12 (0.01–1.02)	−1.31	1.28	0.307	0.27 (0.02–3.31)
Primary	60 (26)	−0.86	0.75	0.249	0.42 (0.09–1.83)	0.53	0.73	0.468	1.69 (0.41–7.08)
Secondary	74 (32)	−0.66	0.65	0.314	0.52 (0.14–1.86)	0.89	0.58	0.120	2.45 (0.79–7.59)
University	76 (33)			Reference				Reference	
**Occupational status**									
Non-employee	47 (20)	−0.18	1.68	0.916	0.84 (0.03–22.76)	2.15	1.53	0.158	8.60 (0.43–171.16)
Employee	54 (24)	1.19	1.51	0.427	3.30 (0.17–63.19)	1.87	1.35	0.164	6.52 (0.46–91.66)
Self-employee	75(33)	1.49	1.49	0.319	4.45 (0.24–83.93)	1.77	1.34	0.185	5.89 (0.43–80.93)
Housewife	44 (19)	0.66	1.56	0.672	1.93 (0.09–41.06)	1.32	1.37	0.336	3.76 (0.25–55.62)
Retired	9 (4)			Reference				Reference	
**Individual monthly income (FCFA** ^**♣**^ **)**									
<50,000	19 (8)	1.23	0.96	0.201	3.43 (0.52–22.63)	0.10	0.92	0.914	1.10 (0.18–6.74)
50,000–100,000	45 (20)	1.25	0.67	0.063	3.49 (0.93–13)	0.07	0.61	0.906	1.07 (0.32–3.56)
100,000–150,000	29 (13)	2.07	0.68	0.002***	7.90 (2.07–30.1)	−0.54	0.86	0.532	0.58 (0.11–3.15)
150,000–200,000	30 (13)	0.16	0.74	0.828	1.17 (0.274–5.04)	−1.09	0.75	0.145	0.33 (0.07–1.46)
≥200,000	106 (46)			Reference				Reference	
**Monthly food expense (FCFA** ^**♣**^ **)**									
<25,000	7 (3)	0.07	1.39	0.962	1.07 (0.07–16.47)	−0.05	1.35	0.969	0.95 (0.07–13.42)
25,000–50,000	27 (12)	−0.38	0.75	0.616	0.69 (0.16–2.99	−0.02	0.77	0.974	0.98 (0.22–4.39)
50,000–75,000	26 (11)	−2.00	0.90	0.027**	0.13 (0.023–0.79)	−0.75	0.81	0.355	0.47 (0.1–2.32)
75,000–100,000	33 (14)	0.661	0.58	0.257	1.94 (0.62–6.07)	1.23	0.60	0.042**	3.43 (1.05–11.25)
≥100,000	136 (60)			Reference				Reference	

** *p < 0.05,*

*** *p < 0.01; %, Percentage; SE, Standard error; OR, Odds ratio*.

♣ *FCFA, Franc de la communauté financière africaine (1 USD = 565.1686 FCFA, https://fr.exchangerates.org.uk/convertir/USD-XOF.html)*.

† *Multinomial regression; Reference group: medium perception*.

*Quality of fit; Pearson Chi square: 49.923, Significance: 0.320*.

As for the “high perception” on the quality and safety of dibiterie meat, it was negatively associated with age (*p* < 0.05) and positively with consumers' monthly food expenditure (*p* < 0.05). Thus, the more people are between 16 and 20 years old, the less strongly they perceive the quality and safety of dibiterie meat (compared to the reference group). In addition, compared to the reference group, people with monthly food expenses of between 75,000 and 100,000 FCFA tend to have a high perception on the quality and safety of dibiterie meat.

### Relationships Between the Variables Linked to the Perception on the Quality and Safety of Dibiterie Meat and the Perception on the Risks of Food Infection

#### Identification of Latent Variables

The PCA allowed to identify the latent variables linked to the perception on the quality and safety of dibiterie meat (Table 5). The perception on the quality of dibiterie meat is described by three latent variables including “expertise of dibiterie,” “price effects,” and “organoleptic quality” with an explained cumulative variance of 95%. The indicators of the perception on the safety of dibiterie meat are grouped around two latent variables, “product safety” and “nutritional quality,” with a cumulative explained variance of about 64%. Moreover, the perception on the risk of food infection is made up of a single factor with an explained variance of about 53%.

**Table 5:** Identification of latent variables of the structural equation model.

**Latent variables**	**Observed variables (Items)**	**Principal component (PC)**		
		**PC 1**	**PC 2**	**PC 3**
**Indicators of perception on the safety of dibiterie meat**				
Expertise of the dibiterie	Dibiterie renown (PQ1)	0.985	0.092	0.098
	Dibiterie name (PQ2)	0.982	0.102	0.104
	Salesperson's expertise (PQ3)	0.974	0.096	0.087
Price effects	Proximity of dibiterie (PQ7)	0.092	0.988	0.042
	Time constraint (PQ6)	0.093	0.972	0.056
	Price of the dibiterie meat (PQ8)	0.098	0.962	−0.005
Organoleptic quality	Taste (after cooking) (PQ4)	0.078	0.016	0.944
	Smell (after cooking) (PQ5)	0.134	0.051	0.935
		KMO index and Bartlett test		
	Kaiser-Meyer-Olkin Index for measuring sampling quality	0.663		
	Bartlett's sphericity test		Chi-square approx. = 3,775.524; df = 28; *p* = 0.000	
		Total variance explained		
	% of variance	36.671	35.978	22.458
	Cumulative %	36.671	72.649	95.107
**Indicators of perception on the safety of dibiterie meat**				
Product safety	Place of animal slaughter (PS1)	0.920	−0.024	–
	Animal slaughter according to the Muslim rite (PS2)	0.913	−0.025	–
	Veterinary stamp (PS3)	0.886	−0.053	–
	Dibiterie hygiene (PS4)	0.338	0.162	–
	Microbes (PS5)	0.330	0.248	–
Nutritional quality	Rich in vitamins (PS6)	0.057	0.917	–
	Rich in energy (PS7)	−0.013	0.908	–
		KMO index and Bartlett test		
	Kaiser-Meyer-Olkin Index for measuring sampling quality	0.690		
	Bartlett's sphericity test		Chi-square approx. = 830.273; df = 21; *p* = 0.000	
		Total variance explained		
	% of variance	38.433	25.105	–
	Cumulative %	38.433	63.538	–
**Perception on the risks of food infection**				
Perception on the risks of food infection	Storage temperature of dibiterie meat is important to avoid food infections (PR4)	0.941	–	–
	Proper cooking of dibiterie meat is important to avoid food infections (PR3)	0.940	–	–
	Raw food can contaminate dibiterie meat (PR2)	0.475	–	–
	Hand washing before dibiterie meat consumption is important to avoid food infections (PR1)	0.373	–	–
		KMO index and Bartlett test		
	Kaiser-Meyer-Olkin Index for measuring sampling quality	0.666		
	Bartlett's sphericity test		Chi-square approx. = 548.340; df = 6; *p* = 0.000	
		Total variance explained		
	% of variance	53.352	–	–
	Cumulative %	53.352	–	–

#### Estimation of the Initial Model

**Figure 1:**
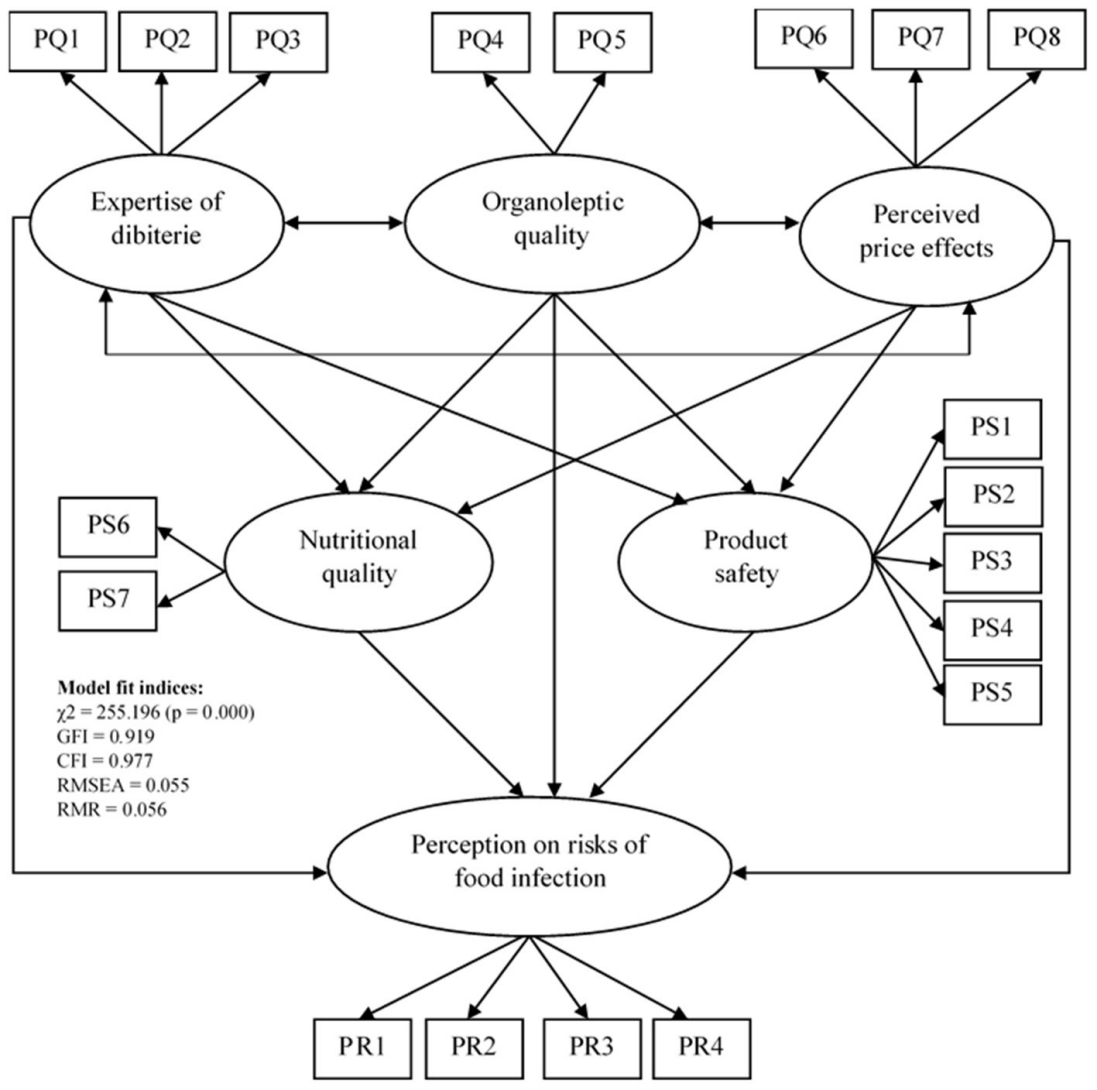
Hypothetical relationships between the latent variables of the perception on the quality, the perception on the safety of dibiterie meat, and the perception on the risks of food infection (PQ, Perception on the quality; PS, Perception on the safety; PR, Perception on the risks).

The initial hypothetical model (Figure 1) deviated significantly from the data according to the strict χ^2^ test [χ^2^ (df = 137, *N* = 291) = 255.196; *p* < 0.01], although it had an acceptable fit according to the approximate fit indices (GFI = 0.919; CFI = 0.977; RMR = 0.056; RMSEA = 0.055). Furthermore, since an overall lack of fit of the model is synonymous with bias in the estimates of the individual parameters, the structure of the model was therefore modified, to obtain a satisfactory fit before proceeding to the examination of the individual estimates.

#### Estimation of the Final Model

**Figure 2:**
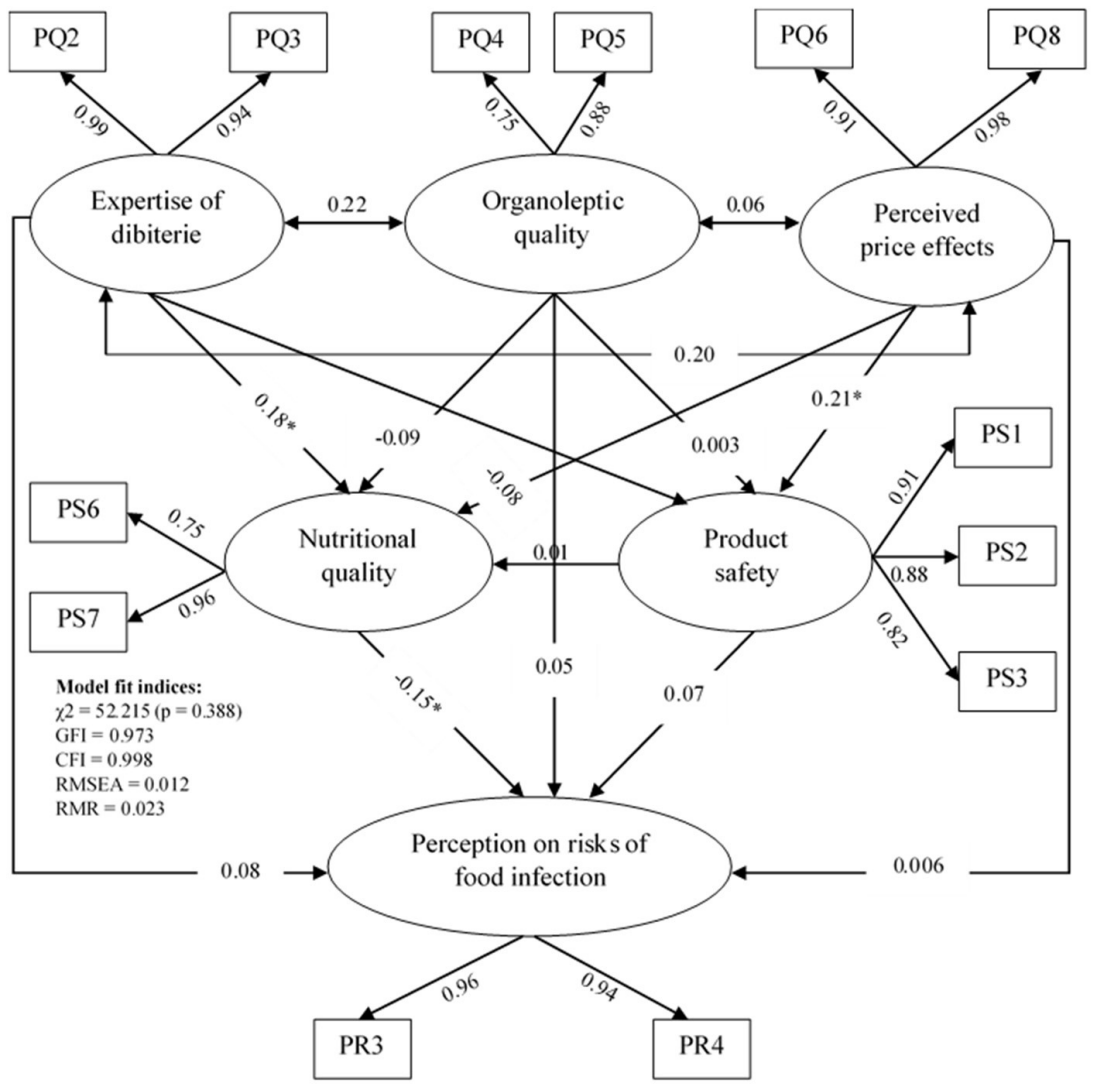
Estimates of the final standardized model of the relationships between the latent variables of the perception on the quality, the perception on the safety of dibiterie meat, and the perception on the risks of food infection (PQ, Perception on the quality; PS, Perception on the safety; PR, Perception on the risks) (*n* = 291). *The value of standardized coefficients that are significant.

The estimates following the respecification of the construct show a good fit between the final model (Figure 2) and the data according to the strict χ^2^ test [χ^2^ (df = 50, *N* = 291) = 252.215; *p* > 0.05]. The fit indices also indicate that the overall fit of the final model was acceptable (RMSEA = 0.012; GFI = 0.973; CFI = 0.998; RMR = 0.023).

#### Product Safety, Nutritional Quality, and Risks of Food Infection

The estimate showed that the perceived price effects was positively associated to the product safety (β = 0.21; *p* < 0.001), while the expertise of the dibiterie had a direct impact on the nutritional quality of the product (β = 0.18; *p* < 0.01). This means that the perceived price effects and the expertise of the dibiterie, respectively, increase the perception on the product safety and the perception on the nutritional quality of the product. Moreover, among the variables tested, only nutritional quality was negatively associated with the perception on the risk of food infection (β = −0.15; *p* < 0.05) (Figure 2). Therefore, the more the nutritional quality of the dibiterie meat is perceived, the less the risk of food infection is perceived.

### Consumer Representations Toward the Risks Associated With the Consumption of Dibiterie Meat

#### Preferences and Incentives Related to the Consumption of Dibiterie Meat

Investigations carried out among consumers and dibiterie tenants indicate that dibiterie meat is consumed because of these nutritional, therapeutic, and organoleptic properties. However, these virtues depend on the species and the age of the animal consumed. Indeed, consumers agree that dibiterie meat prepared from goat meat and lamb meat are the most nutritious; while the meat of an adult sheep is less tender, difficult to digest and can be a source of lower profit for the seller. The words below illustrate these statements:

“*Goat meat gives power, especially the testicles give men sexual power. Moreover, it is said that the men who work in the slaughterhouse (SERAS) love girls, this is due to the meat they consume every day*.” FGD, woman consuming dibiterie meat, Pikine.

“*Meat from young animal is more useful than meat from old animal, it is more productive, it gives you strength. That is why the Moor does not eat the meat of an old beef or an old sheep. He eats lamb or goat meat*.” FGD, male consumer of dibiterie meat, Pikine.

“*In a dibiterie, you have to sell lamb meat, because it is more tender. If you sell meat from adult mutton, it is tough and if a customer eats it, he will not want to come back in your dibiterie. Therefore, there will be a double loss: the non-profit because you are going to buy the sheep and you are not going to benefit, but also it means that the customer will not come back anymore*.” Semi-structured interview with a dibiterie tenant in Pikine.

Consumption of dibiterie meat is also incited by social pressure and the expertise of the dibiterie. Indeed, social pressure, affinity with the seller, and the renown or expertise of the dibiterie are the incentives for buying and consuming dibiterie meat. These different factors are described below by the different actors surveyed.

“*Sometimes my wife tells me she wants meat so that I go to a dibiterie to buy braised meat*.” FGD, male consumer of dibiterie meat, Pikine.

“… *yet I left dibiterie near my workplace, but I came here because it is my favourite dibiterie, because the salesperson masters his activity, also he is open and warm*.” FGD, male consumer of dibiterie meat, Pikine.

“*The communication or the publicity which one makes of me makes me gain customers. So much so that the other sellers of the dibiterie meat think that I wear Talisman; but the secret is at the base linked to my knowledge*.” Semi-structured interview with a dibiterie tenant in Pikine.

#### Representation of the Risks Associated With the Consumption of Dibiterie Meat

According to the consumers of dibiterie meat surveyed, adult beef, cow and mutton are sources of non-communicable diseases, including hypertension and hypotension. The following comments from a consumer consolidate this argument:

“*Eating old mutton or beef is not good for your health. It causes hypertension or hypotension. That is why when I go to a dibiterie I always ask for lamb meat. If a dibiterie doesn't make lamb, I don't buy there*.” FGD, male consumer of dibiterie meat, Pikine.

For those surveyed, hanging meat in the open air in dibiteries does not constitute a risk for the consumer. It allows the buyer, on the other hand, to assess the quality (freshness) of the meat. In fact, according to them, the cooking temperature of meat and faith in God help eliminate pathogenic microorganisms in meat and protect the consumer. The various comments below from customers describe this finding:

“…* of course, the meat is hanging in the open air, but it allows me to know if the meat is still good and has not lasted too long. In addition, it is the fire that kills germs, every germ dies with fire, microbes cannot withstand 37°C*.” FGD, woman consuming dibiteries meat, Pikine.

“*At Thié, it's in the open air, but when a meat is braised, it will drive out germs, but it is God who protects us. We must pronounce the name of God while eating, especially as a Muslim. You surrender to God. You have to be positive in matters of food. We believe in God and we have confidence in him, even a poisoned diet, we will say Bismillah*.” FGD, male consumer of dibiterie meat, Pikine.

In addition, the consumers investigated are aware of the health risks that clandestine slaughtering can engender for human health. Indeed, they argue that dibiterie meat from illegal slaughter is not safe for human consumption because of the diseases that humans can contract through animal products. This statement is described through the comments below from a consumer:

“*I have my special dibiterie, the meat sold there comes from the slaughterhouse. I don't buy from dibiteries that slaughter animals illegally. Because someone can sell a sick sheep to a dibiterie and if you eat this meat, you will get sick too. But at the slaughterhouse there is more security with a vet's stamp. I vigil over the place where the animal is slaughtered. I don't trust the others*.” FGD, woman consuming dibiterie meat, Pikine.

## Discussion

The present study has shown that consumers of dibiterie meat can be classified into three groups according to their level of perception of quality and safety, including low, medium, and high perception. More than half of the consumers surveyed (70%) had medium perception on the quality and safety of dibiterie meat, while individuals with low and high perceptions each represent only 15% of the whole participants. This low proportion of consumers who highly perceive the quality and safety of dibiterie meat may be linked to the difficulty of accessing information on the product that can be used to assess its quality. Indeed, the study showed that consumers rely mainly on the attributes of the experienced quality (taste), extrinsic quality linked to the production environment (dibiterie hygiene, salesperson' expertise, dibiterie renown, and name of the dibiterie), and belief quality (rich in vitamins) to assess the quality of dibiterie meat. According to Grunert (43), when buying and consuming a food product, consumers select, organize, and interpret information for immediate decision-making. Thus, the purchase decision is directly linked to the stimuli available to the consumer before a purchase (26). In addition, faced with the multiple decisions that must be made, most of the indicators that consumers look for in food products are characteristics of experience or credence (belief) that are unknown at the time of purchase (43). Consumers therefore try to reduce this uncertainty by drawing on their own past experience and on information provided by sellers and, to a lesser extent, from the third parties. The exact aspect of this information gathering process and how it leads to decisions depends on the retail environment in which the purchases take place (44). Thus, the ability to assess quality may first and foremost be conditioned by the ability of consumers to read and interpret information on verifiable qualitative attributes (45). Therefore, higher skill levels may lead to more information seeking and better buying results, but that information seeking in some cases can also increase perceived risk and decrease enjoyment and satisfaction (44).

Compared to the reference group (medium perception), the factors associated with low consumer perception on the quality and safety of dibiterie meat were income and food expenditure. Indeed, the income of between 100,000 and 150,000 FCFA/month positively affects the low perception on the quality and safety of dibiterie meat. This suggests that people with a monthly income between 100,000 and 150,000 FCFA have a low perception on the quality and safety of dibiterie meat. Moreover, compared to the reference, food expenses of between 50,000 and 75,000 FCFA/month negatively influence the low perception. Thus, people with food expenses of between 50,000 and 75,000 FCFA/month have a lower tendency to weakly perceive the quality and safety of dibiterie meat. In summary, people belonging to the middle- or upper-income class and spending more on their food have a lower tendency to weakly perceive the quality of dibiterie meat. Therefore, we can deduce the importance of the price's factor in assessing the quality and safety of dibiterie meat. This suggests that, in the market place, consumers are sensitive to the price of dibiterie meat and are willing to support the transaction costs associated with the availability and access to information on the attributes of quality and safety. Furthermore, Mamine et al. (45) point out that the relative ability of consumers to perceive information on quality attributes is sometimes at the root of the controversies that characterize their purchasing rationality. Consequently, the latter use trust and reputation to reduce these costs of quality assessment which also follows a controversial schema (45).

The study also showed that compared to the reference group (medium perception), the high perception on the quality and safety of dibiterie meat is negatively associated with the age between 16 and 20 years. In other words, people between 16 and 20 years old do not highly perceive the quality and safety of dibiterie meat. Furthermore, unlike the low perception, high perception is not significantly associated with income, but rather with food expenditure, and the more people have food expenses of between 75,000 and 100,000 FCFA/month, the more they tend to have a high perception on the quality and safety of dibiterie meat. These results can be explained by the fact that young people are, on the one hand, less concerned with issues related to food quality and safety and, on the other hand, have less skills or experience to identify and interpret the available information on the quality and safety attributes. In contrast, people with high food expenditure demand much more from the quality and safety of the food products they consume. As such, it suggests that they are more willing to research and afford the price necessary to gain access to information enabling them to assess the quality of the products purchased.

We found that the perceived price effects had a significant and positive relationship with the perception of product safety, but had no direct impact on the perception on the risk of food infection. In other words, the perceived price effects increase the perception on the safety of dibiterie meat. This suggests that consumers believe that expensive dibiterie meat provides assurance on the safety of the product. These results are in line with the study by Orou Seko et al. (46) carried out among consumers within the dibiteries. These authors found that consumers surveyed in outlets were willing to pay an extra of $0.5 to $0.84 over the usual selling price of dibiterie meat (between $8.01 and $8.16 per kilogram on average) in order to improve the quality of the product (46). This demonstrates the link between the price and the sanitary quality of food products already demonstrated by several authors in the literature (14, 15, 43, 44, 47–51).

The expertise of the dibiterie indirectly impacted the perception on the risks of food infection through the variable linked to the perception on the nutritional quality of the product. However, the direct path had no effect on the perceived risks of food infection associated with the consumption of dibiterie meat. Indeed, the results showed that the expertise of the dibiterie increases the perception on the nutritional quality of the dibiterie meat, which, in turn, decreases the perceived risks of food infection. This suggests that consumers of dibiterie meat are aware that the expertise (preparation of the meat) that gives the dibiterie renown could lead to an improvement in the nutritional quality of the dibiterie meat and thus reduce consumer perception on the risks of food infection. It also means that faced with the expertise of dibiterie, consumers pay much more attention to the nutritional quality of the meat than to the risk of food infection. Several studies have shown that cooking methods have significant impacts on the nutritional and sanitary quality of the foodstuffs. Indeed, cooking methods are used to improve the microbiological quality of food, destroy various toxins and other contaminants, and, therefore, increase the safety and shelf life of food. In addition, they have greatly contributed to improving the organoleptic quality by generating the formation of commonly appreciated flavors and textures. Although the benefits of culinary processing are numerous and well-identified, it is obvious that cooking and preservation treatments also sometimes lead to a deterioration in the nutritional quality of foods. Among macronutrients, it is mainly proteins and lipids that are affected by heat treatment (52–56). An investigation on the impact of heat treatments (cooking on a traditional oven using wood fire or charcoal) on the nutritional quality of mutton in the different types of dibiteries (Senegalese, Hausa, and Moor) could be of great interest in providing adequate answers to this problem. This should lead to proposals for recommendations to consumers for better guidance on the choice of processed foods to consume and on the preferred cooking methods.

At the end, this study showed that 16% of consumers strongly perceive the quality and safety of dibiterie meat. In addition, the strong perception of the consumers on the quality and safety of dibiterie meat has been positively associated with their monthly food expenditure, while their age explained it negatively. Furthermore, among the latent variables identified, the perceived price effect and the dibiteries' expertise were positively related to the perception on the safety and the perception on the nutritional quality of the product. The nutritional quality of the product perceived by consumers had negatively impacted their perceived risks of food infection. This study suggests the strengthening of hygiene standards in dibiteries and the awareness of consumers, especially young people, about the potential health risks associated with the consumption of dibiterie meat.

## Data Availability Statement

The raw data supporting the conclusions of this article will be made available by the authors, without undue reservation.

## Ethics Statement

The studies involving human participants were reviewed and approved by the Research Ethics Committee of the University Cheikh Anta Diop (No. 0318/2018/CER/UCAD). Written informed consent to participate in this study was provided by the participants' legal guardian/next of kin.

## Author Contributions

MO, WO, NL, and BB: conceptualization, methodology, visualization, and writing—review and editing. MO: data curation, formal analysis, and writing—original draft. NL: investigation. BB: project administration, resources, and validation. WO: supervision. All authors have read and agreed to the published version of the manuscript.

## Funding

The authors acknowledge support from the DELTAS Africa Initiative (Afrique One-ASPIRE/DEL-15-008). Afrique One-ASPIRE was funded by a consortium of donor including the African Academy of Sciences (AAS) Alliance for Accelerating Excellence in Science in Africa (AESA), the New Partnership for Africa's Development Planning and Coordinating (NEPAD) Agency, the Wellcome Trust (107753/A/15/Z), and the department for international development of the UK government. The results and opinions expressed are not those of the funders.

## Conflict of Interest

The authors declare that the research was conducted in the absence of any commercial or financial relationships that could be construed as a potential conflict of interest.

## Publisher's Note

All claims expressed in this article are solely those of the authors and do not necessarily represent those of their affiliated organizations, or those of the publisher, the editors and the reviewers. Any product that may be evaluated in this article, or claim that may be made by its manufacturer, is not guaranteed or endorsed by the publisher.
